# Comparative sequence analysis of the capsular polysaccharide loci of *Actinobacillus pleuropneumoniae* serovars 1–18, and development of two multiplex PCRs for comprehensive capsule typing

**DOI:** 10.1016/j.vetmic.2018.05.011

**Published:** 2018-07

**Authors:** Janine T. Bossé, Yanwen Li, Roberto Fernandez Crespo, Sonia Lacouture, Marcelo Gottschalk, Rita Sárközi, László Fodor, Maria Casas Amoribieta, Øystein Angen, Katerina Nedbalcova, Matthew T.G. Holden, Duncan J. Maskell, Alexander W. Tucker, Brendan W. Wren, Andrew N. Rycroft, Paul R. Langford, on behalf of the BRaDP1T consortium

**Affiliations:** a Section of Paediatrics, Department of Medicine, Imperial College London, St. Mary's Campus, London, UK; b Groupe de Recherche sur les Maladies Infectieuses du Porc, Faculté de Médecine Vétérinaire, Université de Montréal, Québec, Canada; c Department of Microbiology and Infectious Diseases, University of Veterinary Medicine, Budapest, Hungary; d OVISLAB S.L., Barcelona, Spain; e Department of Microbiology and Infection Control, Statens Serum Institut, Copenhagen, Denmark; f Veterinary Research Institute, Hudcova 70, 621 00 Brno, Czech Republic; g The Wellcome Trust Sanger Institute, Hinxton, UK; h Department of Veterinary Medicine, University of Cambridge, Cambridge, UK; i Faculty of Infectious & Tropical Diseases, London School of Hygiene & Tropical Medicine, London, UK; j Department of Pathology and Pathogen Biology, The Royal Veterinary College, Hawkshead Campus, UK

**Keywords:** Diagnostic, mPCR, Capsule typing, *A. pleuropneumoniae*, Serovars

## Abstract

Problems with serological cross-reactivity have led to development of a number of PCRs (individual and multiplex) for molecular typing of *Actinobacillus pleuropneumoniae*, the causative agent of porcine pleuropneumonia. Most of these assays were developed for detection of specific amplicons within capsule biosynthetic genes before the availability of complete sequences for the different serovars. Here we describe comparative analysis of the complete capsular loci for all 18 serovars of *A. pleuropneumoniae*, and development of two multiplex PCRs for comprehensive capsule typing of this important pig pathogen.

## Highlights

• Analysis of complete capsule loci in all 18 serovars of *A. pleuropneumoniae*.
• Novel insights into evolution of capsule loci in *A. pleuropneumoniae*.
• Development of two mPCRs for comprehensive capsule typing.

## 1 Introduction

Pleuropneumonia is an economically important disease causing considerable losses in the worldwide swine industry (Sassu et al., 2017). The causative agent, *Actinobacillus pleuropneumoniae*, can be differentiated into 2 biovars based on the requirement for nicotinamide adenine dinucleotide (NAD-dependent biovar 1; NAD-independent biovar 2); and subsequently into 18 serovars based on surface polysaccharides, mainly capsule (Bossé et al., 2018). The ability to discriminate between serovars is advantageous, as there are differences in geographical distribution that are not static (Gottschalk, 2015; Sassu et al., 2017), as well as differences in levels of virulence (Klitgaard et al., 2010). Thus, accurate typing is essential for diagnosis and for tracking the emergence of serovars rarely, or not previously, reported within a geographical region.

Although a number of serological tests are available for typing *A. pleuropneumoniae* isolates [see (Gottschalk, 2015) for a recent review], the need for high quality reference antisera limits the number of laboratories able to perform diagnostics, and even then, problems with cross reactivity between certain serovars are unavoidable. Increasingly, laboratories are using molecular typing methods to more accurately and reproducibly identify *A. pleuropneumoniae* isolates (Gottschalk, 2015; Sassu et al., 2017). PCRs have been developed for detection of specific CPS genes in most of the currently recognized 18 serovars (except 4, 9, 11, 13 and 14), either individually or in multiplex reactions, for detection of predominant serovars in a given geographical region (Angen et al., 2008; Bossé et al., 2014, 2017, 2018; Ito and Sueyoshi, 2015; Jessing et al., 2003; Schuchert et al., 2004; Turni et al., 2014). Some of these PCR assays were developed prior to the availability of whole genome sequences (wgs), and were based on (sometimes incomplete) sequences of the CPS biosynthetic loci.

The aim of this study was to comprehensively analyze the complete CPS loci for all known serovars of *A. pleuropneumoniae*, and to develop multiplex PCRs capable of their specific identification.

## 2 Materials and methods

### 2.1 *A. pleuropneumoniae* isolates used in this study

**Fig. 1:**
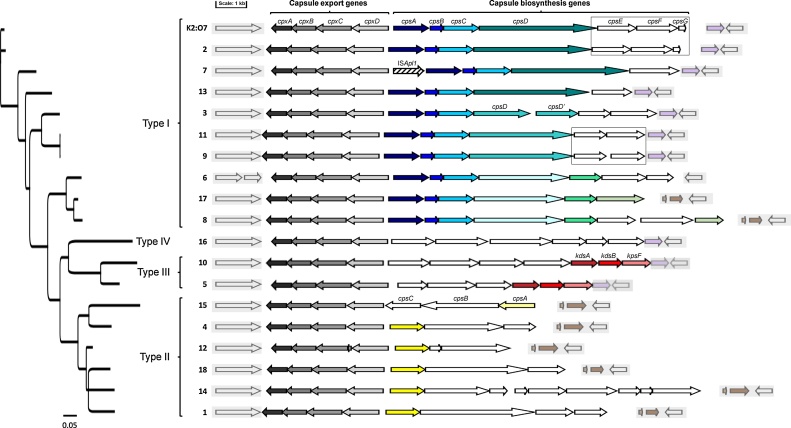
Schematic comparison of the complete capsule loci of *A. pleuropneumoniae* serovars 1–18 and K2:O7. The capsule loci are arranged according to phylogenetic similarity, as indicated by the tree on the left, and are clustered into their respective CPS types (I–IV) as indicated by the labeled brackets. All loci are flanked by the *modF* gene at the start (white arrow in shaded grey box; note an internal stop codon is present in the serovar 6 *modF* sequence), and *ydeN* at the end, preceded either the by the 552–555 bp hypothetical gene or the 114 bp hypothetical and partial *lysA* genes (white arrow, preceded by either mauve or brown arrows, respectively, in shaded grey box). The capsule export genes, *cpxABCD*, are indicated as reverse oriented arrows shaded black to light grey, respectively. The genes of the respective serovar CPS biosynthetic loci are named as follows: *cps2ABCDEFG* (for both K2:O7 and serovar 2); *cps7ABCDE*, *cps13ABCDE*, *cps3ABCDD’EF*, *cps11ABCDEF*, *cps9ABCDEF*, *cps6ABCDEFG*, *cps17ABCDEF*, *cps8ABCDEFGH*, *cps16ABCDEF*, *cps10ABCD kdsAB kpsF*, *cps5ABC kdsAB kpsF*, *cps15ABC*, *cps4ABC*, *cps12ABB’*, *cps18ABC*,*cps14AB1B2B3CDEFG*, *cps1ABCD*. The core *cpsABC* genes conserved in all of type I CPS loci are indicated as the dark, medium, and light blue arrows, respectively (note the extra gene at the start of the serovar 7 biosynthetic locus, shown as a striped arrow, indicates the ISApl*1* insertion present in the AP76 strain, and is not part of the biosynthetic locus). The *cpsD* genes in the type I loci are indicated in different shades of teal, according to similarity greater than 50% identity (note in serovar 3, an internal stop codon has resulted in two orfs, *cps3D* and *cps3D’*). In serovars 6, 17 and 8, the *cpsE* genes share >80% identity and are shown as bright green arrows; and the last gene in the serovar 8 locus, *cps8H*, shares 94% identity with the C-terminal half of the serovar 17 *cpsF* gene, as indicated by the olive shaded arrows in the respective loci. The core genes in the type III CPS loci (*kdsAB* and *kpsF*) are indicated by the dark red, bright red, and pink arrows, respectively, at the ends of the serovar 5 and 10 loci. In the type II loci, the conserved core *cpsA* gene is shown as a yellow arrow. The white arrows in each biosynthetic locus indicate genes unique to each serovar. As expected, the K2:O7 CPS locus shares 96% identity across the entire sequence with that of serovar 2 (with nucleotide differences being mainly found in the *cpsABC* genes), and the specific *cps2EFG* genes found at the ends of both of these loci are boxed. Serovars 9 and 11 share 99% identity across their *cpsEF* genes (also shown boxed), with only a single nucleotide difference resulting in an alternate start codon for the *cpsF* gene in each locus. The Neighbour-Joining tree shown at the left of the figure was constructed using the Tamura-Nei algorithm with 1000 bootstraps, and the width of the line underneath it shows a 5% nucleotide difference (For interpretation of the references to color in this figure legend, the reader is referred to the web version of this article).

All sequences used in this study are shown in Table 1, with accession numbers shown for previously published whole genomes and for the full CPS loci of serovars lacking full genome sequences in Genbank. In this study, wgs data was generated for the reference strains of serovars 13 (N273), 14 (3906), and 15 (HS143), and for two isolates of K2:O7 (7317/84 and 9712534), as previously described (Bossé et al., 2018). The regions of whole genome sequences containing the complete CPS loci (export and biosynthetic genes) were identified initially by using tBLASTn (http://blast.ncbi.nlm.nih.gov /Blast.cgi) to identify the *cpxD* gene (accession AIA09380) common to all *A. pleuropneumoniae* serovars. The complete CPS loci, found between the genes *modF* and *ydeN* in all serovars, were extracted for further analysis using BLASTn and BLASTx. Multiple sequence alignments were performed using ClustalW, and a schematic representation of each locus was generated using Gene Graphics (Harrison et al., 2017), with a Neigbour-Joining tree constructed using the Tamura-Nei algorithm with 1000 bootstraps (Fig. 1).

**Table 1:** Accession numbers for APP sequences used in this study.

Serovar	Strain	Accession	Reference
1	4074^a^	ADOD00000000	Xu et al. (2010)
1	KL 16	CP022715	Park et al. (2017)
2	4222	ADXN00000000	Zhan et al. (2010)
2	S1536	ADOE00000000	Xu et al. (2010)
K2:07	7317/84	MG868951	This study
K2:07	9712534	MG868952	This study
3	JL03	CP000687	Xu et al. (2008)
4	M62	ADOF00000000	Xu et al. (2010)
5b	L20	CP000569	Foote et al. (2008)
6	Femø	ADOG00000000	Xu et al. (2010)
7	AP76	CP001091	
7	S8	ALYN00000000	Li et al. (2012)
8	MIDG2331	LN908249	Bossé et al. (2016)
9	CVJ13261	ADOI00000000	Xu et al. (2010)
10	D13039	ADOJ00000000	Xu et al. (2010)
11	56153	ADOK00000000	Xu et al. (2010)
12	1096	ADOL00000000	Xu et al. (2010)
13	N273	MG868947	This study
14	3906	MG868948	This study
15	HS143	MG868949	This study
16	A-85/14	MG868950	This study
17	16287-1	MG780416	This study
18	7311555	MG780423	This study

The genome listed as serovar 13 str. N273, accession number ADOM00000000, is actually a serovar 7 isolate.

a The genome listed as serovar 1 str. 4074, accession number AACK00000000, is actually a serovar 5 isolate.

The complete capsule loci (with flanking sequences *modF* and *ydeN*) for the reference strains of serovars 13–16, and for the two isolates of K2:O7 (7317/84 and 9712534) have been deposited in GenBank under accession numbers MG868947 to MG868952.

### 2.2 Diagnostic PCRs

The primer pairs used in APP-mPCR1 for specific detection of serovars 1–12 and 15, along with the common *apxIV* amplicon used in previous mPCRs for species level detection of *A. pleuropneumoniae*, are shown in Table 2. Some of the primer pairs were used in our previous mPCR (Bossé et al., 2014), whereas new pairs were designed, either to improve specificity for previously tested serovars (e.g. 3, 6, 7, and 8), or for serovars not previously included in our mPCR. All primers were designed to amplify sequences specific to the relevant serovar, with generation of amplicons of different sizes to allow sufficient separation of all amplicons by gel electrophoresis in 1.5% agarose. The specificity of all primers were initially tested in individual PCRs using genomic DNA from the homologous serovar reference strains, followed by incorporation of all primers into multiplex format, using the Qiagen Multiplex PCR Plus kit as previously described (Bossé et al., 2014). APP-mPCR1 was then tested with all of the 18 *A. pleuropneumoniae* serovar reference strains (i.e. 4074^T^, 1536, S1421, M62, K17, L20, Femø, WF83, 405, CVJ13261, D13039, 56153, 8328, N-273, 3906, HS143, A-85/14, 16287-1, and 7311555, respectively), as well as a set of clinical isolates comprising 2–5 isolates of each of the 18 *A. pleuropneumoniae* serovars, and 31 other porcine-associated bacterial species, used in previous studies (Bossé et al., 2017; 2018). Furthermore, as our previous mPCR (Bossé et al., 2014) was found to detect both serovar 2 and 8 amplicons using DNA from the K2:O7 isolates 7317/84 and 9712534, we specifically compared our new APP-mPCR1 to our previous mPCR for detection of specific amplicons from these two isolates.

**Table 2:** APP-mPCR1 primers for detection of serovars 1–12 and 15.

Primer name	Sequence	Target gene^a^	Amplicon size	Reference or source
AP1F	CTGGAGTAATTACGGCGACTATTCC	*cps1B*	959	Bossé et al. (2014)
AP1R	AGGAGAAGCTAGTAGTACTTGCATTTTC	*cps1B*		
AP2F	GAGTGTGATGATGATGCTCTGGTTC	*cps2E*	247	Bossé et al. (2014)
AP2R	TACCAATAACTGTTGCAACTAACGC	*cps2E*		
AP3F	TTGTAGAGCCCGCCAGATTTACG	*cps3F*	500	This study
AP3R	CATTCGCACCAGCAATCACC	*cps3F*		
AP4F	CAGCATGGGTTTGGTCCTGTTG	*cps4B*	204	This study
AP4R	GGCTTTCTCCGTGTATGAATAAAGTG	*cps4B*		
AP5F	AGCCACAAGACCCGAATGGTATAATG	*cps5B*	825	Bossé et al. (2014)
AP5R	CCATCAAATGCAGCTTCAAGGAGC	*cps5B*		
AP6F	TGACTGGCTTCGTGAAAATGAG	*cps6F*	718	This study
AP6R	GTCTGAAGTTTTATTCGCAGCTCC	*cps6F*		
AP7F	TTGGAATGGATTCATGATTGGGC	*cps7E*	601	Bossé et al. (2014)
AP7R	CGGAAATGGCCTATTGAAAAACG	*cps7E*		
AP8F	ACATCCAAGCCGTTCTCCAG	*cps8F*	1126	This study
AP8R	CATCCATGAGCCAATGAGGG	*cps8G*		
AP9F	GTAGGACGTGGTAACATTGAGGC	*cps9E*	2105	This study
AP9R	ACGGGTGCAATTTCTAAAGCTG	*cps9F*		
AP10F	GGTGGTGATGGAACAAGGTTATGG	*cps10A*	183	Bossé et al. (2014)
AP10R	CTGTAATTGATGCGAAATAGTAGATTGGTGC	*cps10A*		
AP12F	TAAAGGTATTATAACGCCGGCTCT	*cps12A*	347	Bossé et al. (2014)
AP12R	CTCCCATCTGTTGTCTAAGTAGTAG	*cps12A*		
AP15F	GCAACTTGGAGAACATGGTTAAATCAAG	*cps15B*	1595	This study
AP15R	CAACCCTCCAATGTAAGCGAAGG	*cps15C*		
apxIVA1	TTATCCGAACTTTGGTTTAGCC	*apxIV*	418	Zhou et al. (2008)
apxIVA3	CATATTTGATAAAACCATCCGTC	Intergenic^b^		

a For each serovar capsule locus, the biosynthetic genes are labeled alphabetically starting from A in each case.

b Intergenic region immediately downstream of *apxIVA*.

The primer pairs used in APP-mPCR2 for specific detection of *A. pleuropneumoniae* serovars 13–14, and 16–18, along with the species specific *apxIV* amplicon and the full length *nadV* gene found in biovar 2 isolates, are shown in Table 3. The specificity of these primers was evaluated as for the mPCR1 primers above.

**Table 3:** APP-mPCR2 for detection of serovars 13, 14, and 16–18.

Primer name	Sequence	Target gene^a^	Amplicon size	Reference or source
AP13F	GTTGTGTATCGAGGTTGGCATTTC	*cps13E*	665	This study
AP13R	ATGTAAAGGATCTAAGCCGTGTG	*cps13E*		
AP14F	TGCATTACGCTTATATTCTGAATGG	*cps14G*	1911	This study
AP14R	TTGTCGATCGAGAGGGAGTAACG	*ydeN*		
AP16F	TTACTCACTTGGGCTAGGGATAG	*cps16C*	212	Bossé et al. (2017)
AP16R	ACCAGCAATATGATTACGCCC	*cps16D*		
AP17F	TTGTAATGGCGGTGTAATGCTAC	*cps17F*	302	Bosse et al. (2018)
AP17R	CATAAGTGCAGCCATCTCTTTCAG	*cps17F*		
AP18F	CGGAGTTTGGCAGCATAAAGG	*cps18B*	514	Bosse et al. (2018)
AP18R	CCATAATCGGTGCTCAACTAAGAATG	*cps18B*		
nadVF	CTCACTAAACAAAACTCTGCGTTC	*nadV*	1339	This study
nadVR	TTCGGATGACAGAACTTTTACCCG	*nadV*		
apxIVA1	TTATCCGAACTTTGGTTTAGCC	*apxIV*	418	Zhou et al. (2008)
apxIVA3	CATATTTGATAAAACCATCCGTC	Intergenic^b^		

a For each serovar capsule locus, the biosynthetic genes are labelled alphabetically starting from A in each case.

b Intergenic region immediately downstream of *apxIVA*.

## 3 Results and discussion

Initial analyses of the CPS loci in publically available genomes of *A. pleuropneumoniae* (Table 1) revealed that sequences previously deposited in Genbank as Shope 4074 (accession AACK00000000) and N273 (accession ADOM00000000) appear to be incorrect, having CPS loci matching serovars 5 and 7, respectively. The serovar 1 CPS locus is found in the genome of Shope 4074 (accession ADOD00000000), as well as in the recently closed genome of strain KL 16 (accession CP022715) (Xu et al., 2010; Park et al., 2017). The correct serovar 13 CPS locus (accession number MG868947) was identified in the draft genome of N273 that was generated as part of this study, and although similar to the serovar 7 locus (Fig. 1), the *cps13D* gene encodes a predicted protein that shares only 43% identity with that of the Cps7D CDP-glycerol-glycerophosphate glycerophosphotransferase protein. Furthermore, the *cps13E* gene shows no homology to any other sequence in Genbank at the nucleotide level, and the encoded protein shares limited identity (<35%) with hypothetical proteins from various Gram-positive species (e.g. accession number WP_010632822).

Although sequences for the CPS loci of serovars 14, 15, and 16 have previously been deposited in Genbank (accession numbers AB810251, AB701753, KX907602), we have extended these sequences (accession numbers MG868948, MG868949, MG868950) to encompass the complete CPS loci in order to allow a more thorough comparison with the other serovars. Additionally, we have generated draft genomes of two serovar K2:O7 isolates in order to compare their CPS loci (accession numbers MG868951 and MG868952) to those in the two published serovar 2 genomes (accession numbers ADXN00000000 and ADOE00000000) (Xu et al., 2010; Zhan et al., 2010). Although the genes in the K2:O7 CPS loci encode the same proteins as in the serovar 2 CPS loci, there are differences at the nucleotide level that explain amplification of both serovar 2 and 8 amplicons for K2:O7 isolates using our previously designed mPCR (Bossé et al., 2014). In that assay, the serovar 8 primers were designed to amplify an 1106 bp fragment spanning parts of the *cpsAB* genes, and were specific when tested using a large number of clinical isolates, including 45 serovar 2 and 115 serovar 8 isolates (Bossé et al., 2014), however no K2:O7 isolates were tested at that time. Furthermore, we recently found that these serovar 8 primers also produced amplicons from some, but not all, serovar 17 isolates (Bossé et al., 2018). Before redesigning new primers for specific detection of serovar 8, as well as designing primers for detection of specific capsule genes in serovars 4, 9, 11, and 14 (for which there are currently no molecular diagnostics), we thoroughly analysed the complete CPS loci of all known *A. pleuropneumoniae* serovars.

In each serovar, the complete CPS locus is found between the genes *modF*, encoding a putative molybdenum transport ATP-binding protein, and *ydeN*, encoding a predicted serine hydrolase (Fig. 1). The CPS export genes, *cpxABCD*, are transcribed divergently from the CPS biosynthetic genes for all but serovar 15, where the export genes are immediately downstream of the biosynthetic genes, in the same orientation (Ito and Sueyoshi, 2014). The organization of the CPS export genes next to the biosynthetic genes is similar in other members of the *Pasteurellaceae,* as well as in other Gram-negative bacteria, such as *Escherichia coli* and *Neisseria meningitidis,* suggesting a common molecular origin for these loci (Boulnoisl and Jann, 1989; Frosch et al., 1991; Lâm et al., 2011). In many of these bacteria, a third region containing genes involved in post-polymerization modifications/transport is found on the other side of the biosynthetic locus, such that the central biosynthetic genes (region II) are variable, whilst the flanking regions I and III are constant in a given species (Boulnoisl and Jann, 1989; Frosch et al., 1991). In *A. pleuropneumoniae*, only regions I and II are contiguous, whereas genes encoding proteins sharing 63% and 66% identity, respectively, with HcsA and HcsB (encoded by region III of *Haemophilus influenzae*) are found elsewhere in the chromosome (accession numbers WP_039709373 and WP_005610771).

The CPS biosynthetic loci of the different *A. pleuropneumoniae* serovars can be grouped into four types (Fig. 1), with common core genes identified in each for types I-III, and type IV only found in serovar 16 (Xu et al., 2010; Bossé et al., 2017). The most common are the type I loci, found in serovars 2, 3, 6, 7, 8, 9, 11, 13, and 17 (Xu et al., 2010; Bossé et al., 2018), which produce teichoic acid-type polymers with phosphodiester linkages joining repeating glycosyl-glycitol units (Perry et al., 1990; MacLean et al., 2004). The core *cpsABC* genes of the type I loci are well conserved, sharing a minimum of 88% identity, and encode a CDP-glycerol glycerophosphotransferase, a glycerol-3-phosphate cytidylyltransferase, and a protein of unknown function, respectively. Although less well conserved, the type I *cpsD* genes all encode proteins with a glycosyl transferase domain (pfam04464) and a glycosyl transferase group 1 domain (either pfam00534 or pfam13692), that are, along with the products of the *cpsABC* genes, predicted to be involved in synthesis of the teichoic acid-type polymers characteristic of type I CPSs. In serovar 3, the *cpsD* gene has been split into two open reading frames due to an internal stop codon. It is not known if the second orf (*cpsD’*) is translated, as there is no obvious ribosomal binding site preceding the start. The sequences spanning *cpsD* and *cpsD’* in serovar 3 share 86% identity with the *cpsD* genes found in serovars 9 and 11; the *cpsD* gene from serovar 2 shares 76% identity with that of serovar 7, but only 56% with that of serovar 13; and the *cpsD* gene from serovar 8 shares 96% and 98% identity with those from serovars 6 and 17, respectively. Serovars 6, 8 and 17 further share a common *cpsE* gene (84% identity for the serovar 6 gene compared to the serovar 8 and 17 genes, which share 99% identity). Additionally, the last gene of the serovar 8 locus (*cps8H*) shares 90% identity with the final 911 bases of the 1548 bp *cps17F* gene, as previously noted (2).

Type II loci are found in serovars 1, 4, 12, 14, 15, and 18 (Xu et al., 2010; Ito and Sueyoshi, 2014; Ito, 2015; Bossé et al., 2018), and produce repeating oligosaccharide polymers with phosphate linkages, as shown for serovars 1, 4, 12, and 15 (Perry et al., 1990; 2011). The conserved *cpsA* genes in these loci (99% identical for all but *cps15A*, which shares only 65% identity) all encode a capsular polysaccharide phosphotransferase which likely mediates the phosphate linkages characteristic of type II CPSs. Serovars 5 and 10 have type III loci and produce glycosidically linked sugar polymers (Perry et al., 1990), with the common core genes (*kdsA*, *kdsB* and *kpsF,* found at the 3′ end of theses loci) encoding 2-dehydro-3-deoxyphosphooctonate aldolase, 3-deoxy-manno-octulosonate cytidylyltransferase, and arabinose 5-phosphate isomerase, respectively. The serovar 16 CPS locus (type IV) is entirely unique (Bossé et al., 2017), and the structure of this CPS has not yet been determined.

All of the type II loci have a 114 bp orf followed by a partial *lysA* gene (of varying length) upstream of *ydeN* (Fig. 1) that are not likely involved in CPS synthesis. These sequences are also found in the type I loci of serovars 8 and 17; whereas all the remaining serovars with type I, III and IV loci, have a 552–555 bp gene encoding a hypothetical protein of unknown function (though again, not likely involved in CPS synthesis) immediately upstream of *ydeN*, except for the serovar 6 reference strain Femø, where *ydeN* immediately follows the last *cps* gene (Fig. 1). These genes preceding *ydeN* delineate the 3′ boundaries of the different CPS biosynthetic loci in *A. pleuropneumoniae,* and give an indication of possible evolutionary origins.

There are terminal inverted repeats (IRs) of 35–46 bp, containing a central IR sequence, flanking all of the *A. pleuropneumoniae* capsule loci that end with the 114 bp orf and partial *lysA* gene. Along with the atypically low GC content of the *A. pleuropneumoniae* CPS loci (especially the biosynthetic genes that have GC contents around 10% lower than respective genomes), the presence of IRs at the boundaries are indicative of the CPS loci being insertion elements acquired by horizontal transfer (Darmon and Leach, 2014). All *A. pleuropneumoniae* serovars, regardless of capsule type, have at least part of the 46 bp sequence TAAAGGAAATCCCCc/tTCTTTAGTAAAGAGGGg/aTTAGGGGAGATTTG

(lower case letters indicating differences in some serovars at these bases; IR sequence underlined) downstream of *modF*, along with an almost perfect repeat of the central IR (CCCCTCTTTGCTAAAGAGGGG) close to the 3′ end of *cpxA*, suggesting it may be a rho-independent terminator for this gene. In serovars 1, 9, 11, and 18, there may have been recombination between the two copies of the IR such that only a single copy is found, with 35 of the 46 bp sequence conserved (TAAAGGAAATCCCCCTCTTTAGTAAAGAGGGGGGAT) upstream of *cpxA* in these serovars. An almost identical, but inverted, 46 bp sequence (CAAATCTCCCCTATCc/aCCTCTTTACTAAAGAGGGGg/aATTTCCTTTA) is found downstream of the final CPS biosynthetic gene in all loci that are followed by the 114 bp orf and partial *lysA* gene, whereas in serovars with CPS loci followed by a 552–555 bp gene, no evidence of this sequence is seen. These data suggest that, in *A. pleuropneumoniae*, a type II CPS locus was the first to be acquired by horizontal transfer, with subsequent diversification of the other biosynthetic loci via gene deletion and/or acquisition of other capsule genes by horizontal transfer and homologous recombination via conserved flanking sequences.

The *A. pleuropneumoniae* serovar 14 type II CPS locus was previously shown to be almost identical to that found in *Actinobacillus suis* K1 strains such as ATCC 33415 (accession CP009159) (Ito, 2015). As in *A. pleuropneumoniae*, the CPS export genes in *A. suis* are downstream of *modF.* The biosynthetic genes in *A. suis* are followed by the same 114 bp orf as in the *A. pleuropneumoniae* type II CPS loci. However, in *A. suis*, this orf is followed by a complete *lysA* gene (1251 bp), and three further genes (*gst*, *hemN,* and a 600 bp gene encoding a putative nucleoside-diphosphate-sugarepimerase), prior to the *ydeN* gene. This variation in gene arrangement allows differentiation of these two species by diagnostic PCR (see below). In *A. pleuropneumoniae*, the five genes found upstream of *ydeN* in *A. suis* are found elsewhere in the genome (downstream of *frdD*). These data suggest that *A. pleuropneumoniae* serovar 14 may have acquired the *A. suis* CPS locus *en bloc*, with the resulting duplication of the 114 bp orf and part of the *lysA* gene. The other *A. pleuropneumoniae* serovars with type II CPS loci have reduced complexity of CPS genes compared to serovar 14, with only the *cpsA* gene conserved, and the other genes specific for each serovar.

Previously, the most complex mPCR for typing of *A. pleuropneumoniae* contained primers for detection of serovars 1–3, 5–8, 10 and 12, with the addition of primers for *apxIV* to allow detection of other serovars not included (Bossé et al., 2014). Now, with a total of 18 serovars, plus the species-specific *apxIV* amplicon, it was technically challenging to accurately resolve all of the products in a single mPCR. We therefore developed two mPCRs (APP mPCR1 and APP mPCR2), with the first capable of detecting serovars 1–12 and 15, and the second for detection of serovars 13, 14, and 16–18. All primer sequences for APP mPCR1 and APP mPCR2 are shown in Table 2, Table 3. To reduce the risk of non-specific priming, all primers were designed to have a Tm of 58–63 °C.

We kept some of the primer pairs from our previous 9-serovar mPCR (Bossé et al., 2014), including those for detection of serovars 1, 2, 5, 10 and 12, but revised our selection of primers for serovars 3, 6, 7, and 8 in order to target the more serovar-specific genes towards the end of these biosynthetic loci. To maintain good size separation of products in the revised mPCR, the new serovar 3, 6, 7, and 8 primers were designed to produce similar sized amplicons as the previous primer pairs. To this new mPCR (APP mPCR1), we added primers for detection of a 1595 bp fragment spanning the *cpsBC* genes of serovar 15. As there have not previously been diagnostic PCRs for the capsule genes of serovars 4, 9, or 11, we analyzed their biosynthetic loci in order to find appropriate specific priming sites. For serovar 4, primers were designed to amplify a unique 204 bp sequence at the 3′ end of the *cpsB* gene. Alignments of the complete CPS loci (including the export genes and flanking sequences to *modF* and the 555 bp gene) of the serovar 9 and 11 reference strains revealed the only difference is a single base deletion in the final *cps* gene in serovar 11, such that the reading frame ahead of the deletion shifted to use an alternate start codon. In serovar 9, the *cpsF* gene is 1146 bp, and that of serovar 11 is 1242 bp. The encoded proteins have the same C-terminal 349 AAs. It is possible that the altered N-terminal AAs are responsible for the slight differences in the CPS structures of these serovars reported by Perry et al. (Perry et al., 1990). These serovars also share an almost identical LPS O-antigen (Perry et al., 1990), and serologically, it is difficult to distinguish serovars 9 and 11. As these serovars also produce the same complement of Apx toxins (Frey, 1995), there may be little value in distinguishing them. The primers we have designed for combined detection of serovars 9/11 amplify a 2105 bp fragment spanning their *cpsEF* genes.

**Fig. 2:**
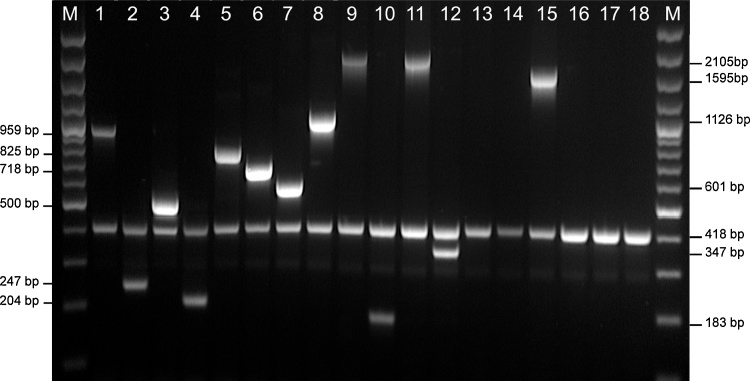
Serovar-specific detection of amplicons from *A. pleuropneumoniae* serovars 1–12 and 15 by APP mPCR1. An *apxIV* (418-bp) amplicon is detected in all 18 serovar reference strains. Lane M contains molecular size markers (100-bp Plus DNA Ladder; Invitogen). Lanes 1 to 18 contain the following strains: 1, 4074 T; 2, S1536; 3, S1421; 4, M62; 5, L20; 6, Femø; 7, WF83; 8, 405; 9, CVJ13261; 10, D13039; 11, 56153; 12, 8329; 13, N-273; 14, 3906; 15, HS143; 16, A-85/14; 17, 16287-1; 18, 7311555.

**Fig. 3:**
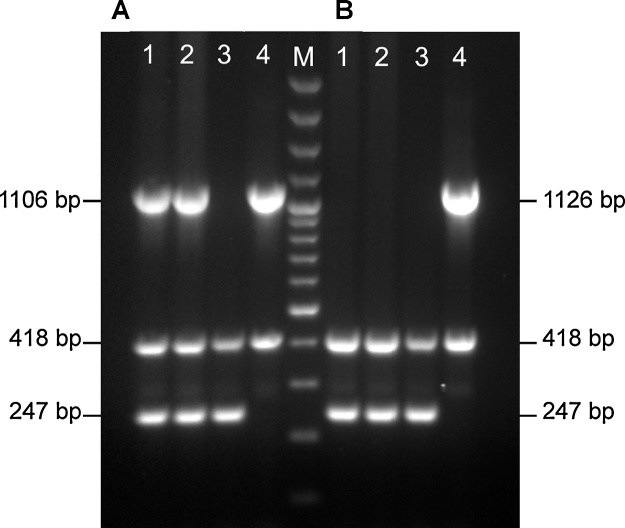
Comparison of previous mPCR (8), and new APP mPCR1 for detection of *A. pleuropneumoniae* serovars 2, 8, and K2:O7. The K2:O7 isolates (7317/84 in lane 1; 9712534 in lane 2) produced amplicons indicative of both serovars 2 and 8 with the previous mPCR (A), but only the correct serovar 2 amplicon with APP mPCR1 (B). Lanes 3 and 4 contain serovar 2 strain S1536 and serovar 8 strain 405, respectively. Lane M contains molecular size markers (100-bp Plus DNA Ladder; Invitogen).

The new APP-mPCR1 detects amplicons from serovars 1–12 and 15, ranging in size from 204 to 2105 bp, plus the 418 bp *apxIV* amplicon as an internal control for detection of all *A. pleuropneumoniae* serovars, as shown for each of the *A. pleuropneumoniae* reference strains tested (Fig. 2). The specificity of this mPCR was further demonstrated using DNA prepared from our collection of clinical *A. pleuropneumoniae* isolates, other *Actinobacillus* species, other *Pasteurellaceae*, and other major pathogens of pigs, in addition to virtual PCRs using all available genomes of the species investigated, where available, as previously described (Bossé et al., 2017). All *A. pleuropneumoniae* isolates produced the 418 bp *apxIV* amplicon in addition to the appropriate serovar-specific bands for each of serovars 1–12 and 15. Furthermore, DNA from the K2:O7 isolates 7317/84 and 9712534, that produced amplicons for both serovars 2 and 8 (*and apxIV*) with our previous 9-serovar mPCR, produced only the serovar 2 and *apxIV* amplicons in the new APP-mPCR1 (Fig. 3). All non-*A. pleuropneumoniae* isolates were negative for all amplicons (data not shown).

For development of the new APP-mPCR2, we combined the same *apxIV* primers used in APP-mPCR1 with our previously designed primers for detection of the most recently identified serovars 16, 17 and 18 (212, 302, and 514 bp amplicons, respectively) (Bossé et al., 2017; 2018). To this mPCR, we added primers for detection of serovars 13 and 14. As mentioned at the beginning of this discussion, the serovar 13 CPS locus contains a unique gene, *cps13E*, not seen in any other sequenced bacterium, so we designed primers to detect a 665 bp fragment of this gene. The analysis of the serovar 14 CPS locus, as discussed above, indicated that primers designed to detect any of the *cps* genes would also detect the related swine pathogen, *A. suis*. However, by designing primers to amplify a 1911 bp fragment from *cps14G* to *ydeN*, it is possible to differentiate this sequence from *A. suis*, where the *ydeN* gene is almost 6 kb downstream of the final *cps* gene. Since serovars 13, 14, and 17 are predominantly biovar 2, with biovar 1 isolates also reported for serovars 13 and 17 (Bossé et al., 2018; Gottschalk, 2015; Sassu et al., 2017), we added primers for detection of a 1339 bp fragment of the *nadV* gene, which confers NAD-independence.

**Fig. 4:**
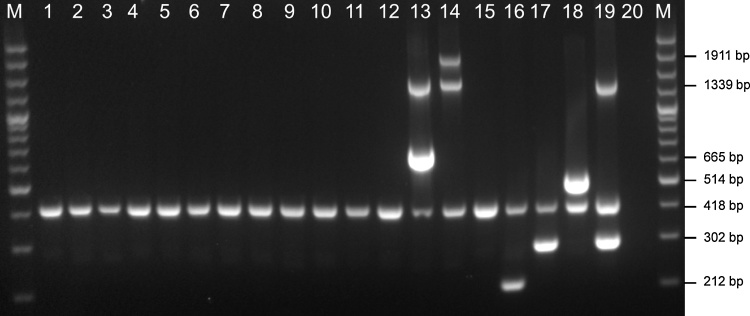
Serovar-specific detection of amplicons from *A. pleuropneumoniae* serovars 13–14 and 16–18 by APP mPCR2. An apxIV (418-bp) amplicon is detected in all 18 serovar reference strains, and an additional 1339 bp nadV amplicon is detected only in the biovar 2 strains. Lane M contains molecular size markers (100-bp Plus DNA Ladder; Invitogen). Lanes 1 to 18 contain the following strains: 1, 4074 T; 2, S1536; 3, S1421; 4, M62; 5, L20; 6, Femø; 7, WF83; 8, 405; 9, CVJ13261; 10, D13039; 11, 56153; 12, 8329; 13, N-273; 14, 3906; 15, HS143; 16, A-85/14; 17, 16287-1; 18, 7311555. Lane 19 contains the biovar 2 serovar 17 isolate, 14-022, and Lane 20 contains A. suis CCM 5586T.

Thus, the new APP-mPCR2 detects amplicons for *apxIV, nadV,* and serovars 13–14 and 16–18, ranging in size from 212 to 1911 bp. Specific detection of these amplicons was demonstrated using DNA from each of the *A. pleuropneumoniae* reference strains (Fig. 4), as well as the same set of samples used above for validation of the APP-mPCR1. Again, all non-*A. pleuropneumoniae* isolates were negative for all amplicons (data not shown). Comparison of biovar 1 and 2 isolates of serovar 17 show that the inclusion of the *nadV* primers in this mPCR facilitate differentiation of these isolates (Fig. 4). Lack of detection of a *nadV* amplicon in NAD-independent non-*A. pleuropneumoniae* isolates, such as *A. suis*, indicates that the primer sequences used are specific for the *A. pleuropneumoniae nadV* gene.

In conclusion, we have developed two mPCRs, APP-mPCR1 and APP-mPCR2, for specific detection of all 18 known serovars of *A. pleuropneumoniae*. Inclusion of primers for detection of *apxIV* in both mPCRs provides an internal control for species-specific detection of all *A. pleuropneumoniae* serovars. In general, we would recommend testing samples with APP-mPCR1 first. Any samples producing a positive *apxIV* band, but none of the amplicons for serovars 1–12 or 15, should then be tested using APP-mPCR2.

## Funding

This work was supported by a Longer and Larger (LoLa) grant from the Biotechnology and Biological Sciences Research Council (grant numbers BB/G020744/1, BB/G019177/1, BB/G019274/1 and BB/G018553/1), the UK Department for Environment, Food and Rural Affairs, and Zoetis awarded to the Bacterial Respiratory Diseases of Pigs-1 Technology (BRaDP1T) consortium. MTGH was supported by the Wellcome Trust (grant number 098051). RS and LF were supported by the Hungarian Scientific Research Fund (OTKA 112826). Genome sequencing provided by MicrobesNG (www.microbesng.uk) was supported by the BBSRC (grant number BB/L024209/1).

## Conflict of interest

We declare that we have no conflict of interest.
